# Spherical Robots for Special Purposes: A Review on Current Possibilities

**DOI:** 10.3390/s22041413

**Published:** 2022-02-12

**Authors:** Marek Bujňák, Rastislav Pirník, Karol Rástočný, Aleš Janota, Dušan Nemec, Pavol Kuchár, Tomáš Tichý, Zbigniew Łukasik

**Affiliations:** 1 Faculty of Electrical Engineering and Information Technology, University of Zilina, 010 26 Zilina, Slovakia; marek.bujnak@feit.uniza.sk (M.B.); karol.rastocny@feit.uniza.sk (K.R.); ales.janota@feit.uniza.sk (A.J.); dusan.nemec@feit.uniza.sk (D.N.); pavol.kuchar@feit.uniza.sk (P.K.); 2 Faculty of Transportation Sciences, Czech Technical University in Prague, 110 00 Prague, Czech Republic; tomas.Tichy@cvut.cz; 3 Faculty of Transport, Electrical Engineering and Informatics, University of Technology and Humanities in Radom, 26-600 Radom, Poland; z.lukasik@uthrad.pl

**Keywords:** spherical robot, mobile robot, inertial sensors, cameras, temperature sensors, gas sensors, LiDAR, special applications, tunnel applications

## Abstract

The review discusses the possibilities of different driving mechanisms and sensors of spherical robots, and a special kind of mobile robots is introduced and discussed. The sensors discussed can expand robots’ sensing capabilities which are typically very limited. Most spherical robots have holonomic characteristics and protect the inner environment using a shell. Today, there are a diversity of driving mechanisms. Therefore, this article provides a review of all of them and identifies their basic properties. Accordingly, many spherical robots have only inner sensors for moving, balancing, driving, etc. However, a few of them are also equipped with sensors that can measure environmental properties. Therefore, in this paper, we propose the possibility of using such sensors as cameras, LiDARs, thermocouples, and gas sensors, which can be used for special purposes underground, for example, in mines, underground tunnels, or road tunnels. After combining all components are combined, it is possible to design a special type of spherical robot designed for underground exploration, such as accidents in mines or road tunnels.

## 1. Introduction

The “special purpose”, in this case, means accidents in the underground space, more specifically, in the road tunnel. During a tunnel accident, various situations can occur, such as leakage of dangerous liquids and gases into the environment, fire, the danger of injury or death of the participants in the traffic accident, huge material damage, and more. Therefore, the fastest and most accurate intervention of the rescue services is needed. However, rescue services are not always able to intervene directly. They would be exposed to risk because they do not know the environment. They need to sufficiently gain knowledge of all the details of the accident.

Based on Reference [1], the authors tried to set the passive sensor in motion on the basis of a certain type of driving mechanism. The first part showed the possibilities of spherical robots to be used in different field of applications (Section 1.1). The survey showed that the considered sensors from the Reference [1] must be placed inside the shell of the spherical robot so that nothing prevents it from smoothing motion. Subsequently, an overview of possible solutions for the control mechanism of the spherical robot (Section 2) was created. This driving mechanism must meet conditions, such as stop on a sloping surface (rising and falling in of a tunnel) and overcoming a step obstacle (sidewalk in tunnels), and, at the same time, it was possible to place sensors on the driving mechanism. In Section 3, an overview was created of inertial sensors, which can be used for a spherical robot for navigation in underground space. Given the sensors that were used in Reference [1], we tried to expand and create an overview of cheap but reliable sensors (Section 4). We focused mainly on measuring gases that are dangerous in accidents in underground and closed spaces (CO and CO${}_{2}$). In addition, the temperature around the spherical robot is necessary to measure because, in an underground space, the temperature can increase immediately. It is also appropriate to use cameras (visible spectrum and IR spectrum) for reconnaissance purposes, so that rescuers do not enter an unsafe environment and, thus, be exposed to some risk. In case the underground space is smoky, or otherwise has reduced visibility, it is appropriate that the spherical robot has LiDAR. This sensor can be used to collect data and then create a 2D map of the space. All these sensors will provide rescue services with a wider range of information that can lead to improved and more effective intervention. The article also includes a section (Section 5) where the authors focused on special applications of mobile robots in the field of surveillance, reconnaissance, fire, search, and rescue. Although this article falls into the category of review, the authors have decided to publish the direction of future research. It connects the sensory part with the driving mechanism and, thus, creates a new perspective on the issue of rescue and reconnaissance robots germane to in-ground space (Section 6), such as the tunnels. By combining these parts, some properties of the spherical robot will be improved. One of the authors, in his PhD work, deals with this issue.

### 1.1. Applications of Spherical Robots

A spherical robot (SR) is a special kind of mobile robot that has a ball’s characteristic and is made up of the steering and the driving mechanism being placed inside of the spherical shell. Most SRs depend on internal weight distribution or the relocation of the center of the mass point. The SR has the character of flexible movement and the spherical shell cover that protects the inner mechanism. In line with this, the SR can be utilized in an environment with dangerous properties, such as in rain or a flammable area.

The development of SRs has recently received significant attention. This type of robot has several advantages, as a of robot of this kind, in comparison with the traditional robots with wheels, belts, or legs [2]:

- the outer shell protects the whole system of the robots,
- the smoothness of movement,
- great power effectivity,
- the motion can be omnidirectional due to the design,
- the possibility of movement on water or throwing a robot, and
- the robot cannot be overturned.

Besides the advantages, SRs also have several disadvantages, such as:

- the small maximum torque,
- problems with movement on uneven and steep terrain,
- limitations in the use of environmental sensors,
- the size of the shell depends on the construction of the inner driving mechanism, and
- complicated design.

The SR can be applied to various fields of application. The first example to be considered is DAEDALUS [3], which will be used to explore the moon caves, or group of rovers equipped with minimal control mechanisms based on design of Tumbleweed dedicated to explore Mars [4]. Similar to drones, SR can also join swarms and move as one whole. An example of such a robot is FreeBOT [5], which is able to join and move as a swarm. Accordingly, these robots can physically join and move as one robot and also can overcome obstacles together. Alternatively, a swarm of SRs might create a sensor network designed to measure ambient temperature and relative humidity [6]. One of the new things is a hybrid SR, having been coupled connected with a drone. Reference [7] puts forward a robot being able to climb, roll, and fly, by combining four-legged crawling, wheel-rolling, and four-rotor-flying movement methods. Similarly, Reference [8] focuses on a robot being capable of rolling on both water surfaces and on ground surfaces and flying in the air by changing the pitch angle of each propeller and adopting corresponding control algorithms. In Reference [9], authors propose a micro spherical rolling and flying robot. In addition, a novel spherical robot, named flying-crawling spherical robot, is designed in Reference [10]. It can fly or roll on the ground with its single rotor and eight tail fins. Some analogical examples of hybrid SRs are swimming or underwater SR. Specifically, Reference [11] provides a description of a new concept of spherical underwater robot propelled by thrust vector synthetic jet actuator. Furthermore, a novel amphibious spherical robot system with Jacobian matrix kinematics verification is proposed in Reference [12]. Authors in Reference [13] came up with a legged, Multi-Vectored Water-Jet Composite Propulsion Mechanism (LMVWCPM) designed with four legs, one of which contains three connecting rod parts, one water-jet thruster, and three joints driven by digital servos. Lastly, Reference [14] introduces system able to track the color coded pattern simultaneously sending feedback on the orientation command to the on board controller to achieve tracking and following of a given target underwater. References [15,16] deal with SRs which were interconnected and used as wheels for an omnidirectional chassis. SRs can be used also for exploration, as well as for protection, of large areas or rooms [17]. In Reference [18], the hardware division of a technological solution for child monitoring outlined development of a semi-autonomous spherical robot to follow a subject’s movements and moves throughout the room. Reference [19] presents a spherical robot carrying a binocular stereo vision, referred to as VisionBot. The robot aims to use stereo vision to enhance the environmental perception of spherical robots, thus reducing cumulative errors caused by sliding, mechanical clearance, and model error. Authors in Reference [20] give description of a computer vision system consisting of several spherical mobile devices with a digital camera and microcomputer inside. Another field where SRs can be used is children’s toys [21].

The motion of SRs must be described in terms of the kinematics and dynamics of their behavior. Hence, the following references comprise detailed descriptions of some of them. Reference [22] presents a large-size spherical robot used for polar scientific research, which can drive by utilizing the wind. In case the wind is strong, the wind drives the robot, and the inside instrument is intended for adjustment of the direction. On the contrary, if the wind speed low, it can move and steer by inertia drive though the pendulum. Authors in Reference [23] are exploring the derivation of the Lagrangian for different configurations of spherical robots, taking Euler Lagrange equations and additional constraints for the generalized velocities into consideration. The degrees of freedom are specified with rotation matrices and linear displacement vectors, thus allowing to firstly determine the necessary elements for all the models in a procedural way and, secondly, to obtain the dynamics of the system. One of the models obtained in the way mentioned above is used for simple velocity control tests with two approaches, using the torque and velocity of the rotatory actuators as control inputs. As pointed out in Reference [24], an internal device’s climbing up the internal surface of the shell results in a gravity-powered pendulum effect, propelling the robot forward up to a speed of 0.16 m/s. Reference [25] is considering the motion of a spherical robot with periodically changing moments of inertia and gyrostatic momentum. Equations of motion are derived within the framework of the model of “rubber” rolling (without slipping and twisting). Authors in Reference [26] derive a dynamic model of a spherical rolling robot having new driving mechanism equipped with a gyro and design a controller to stabilize a desired translational motion of the robot. Due to the angular momentum of the gyro, nutation motion of the internal mechanism of the robot might be caused.

## 2. Driving Mechanism

Generally, driving mechanisms can be divided into three main types, in terms of the used driving method:

- direct driving method,
- gravity driving method, and
- angular momentum driving method.

In general, correct design of the drive mechanism, as well as design of the controller, plays a pivotal role for correct control of the outer shell of SRs. Despite the fact that authors of References [2,27] have offered the overview of driving mechanisms of SRs, both publications only cited articles and research published up until 2012. For this reason, the following part of the article deals with an updated overview of all the driving mechanisms that have been preferably indexed in the WoS, Scopus, and IEEE databases, up to 2021.

### 2.1. Kinematics of a Spherical Robot

In general, the spherical robot has 3 degrees of freedom: two translational and one rotational. If we assume that the robot is moving on a planar surface, the translational coordinates are cartesian (x,y), and the translational velocity is $v=(v_{x},v_{y})$. The horizontal orientation of the robot can be described by heading angle ψ. If we assume that the robot is not sliding, the same 3 degrees of freedom can be understood as arbitrary rotation in 3-dimensional space, quantified by the angular velocity vector $\omega =(\omega_{x},\omega_{y},\omega_{z})$. Please note that the angular velocity components are measured with respect to the global coordinates. The horizontal part of the angular velocity $\omega_{xy}=\left(\omega_{x},\omega_{y},0\right)$ correlates with the translational velocity:

(1) $$v=\omega \times r=-\omega_{xy}\times \left(0\;0\;R\right)=\left(-\omega_{y}R\;\omega_{x}R\;0\right).$$

The vertical part of the angular velocity simply corresponds to the rate of the heading (yaw) angle:

(2) $$\frac{d\psi }{dt}=\omega_{z}.$$

The orientation of the spherical robot (attitude) can be described by Euler angles. There are 12 possible conventions of them, but we use the Z-Y-X convention, widely used in aeronautics, and often referred to as Roll-Pitch-Yaw convention. Using this convention, along with **NED** (x-North, y-East, z-Down) axis orientation, the global angular velocity can be computed from the local angular velocity of the robot [28] (measured by an on-board gyroscope mounted on outer sphere):

(3) $$\omega =\underset{T}{\underset{︸}{\left(\begin{array}{ccc} c_{\Theta }c_{\psi } & s_{\varphi }s_{\Theta }c_{\psi }-c_{\varphi }s_{\psi } & c_{\varphi }s_{\Theta }c_{\psi }+s_{\varphi }s_{\psi }\\ c_{\Theta }s_{\psi } & s_{\varphi }s_{\Theta }s_{\psi }+c_{\varphi }c_{\psi } & c_{\varphi }s_{\Theta }s_{\psi }-s_{\varphi }c_{\psi }\\ -s_{\Theta } & s_{\varphi }c_{\Theta } & c_{\varphi }c_{\Theta } \end{array}\right)}}\cdot \left(\begin{array}{c} \omega_{x}\\ \omega_{y}\\ \omega_{z} \end{array}\right),$$

where φ,Θ,ψ are roll, pitch, and yaw (Euler angles), respectively, and $s_{\varphi }=sin\varphi ,c_{\Theta }=cos\Theta$, etc.

The goal of the robot’s propulsion is to cause the robot’s rotation. Possible actuation principles are described below.

### 2.2. Direct Driving Method

The direct driving method is designed in a simple manner. This mechanism transmits the motor torque directly to the outer shell as the driving force of the robot. This method requires a specially designed mechanism.

#### 2.2.1. Single Wheel

**Figure 1:**
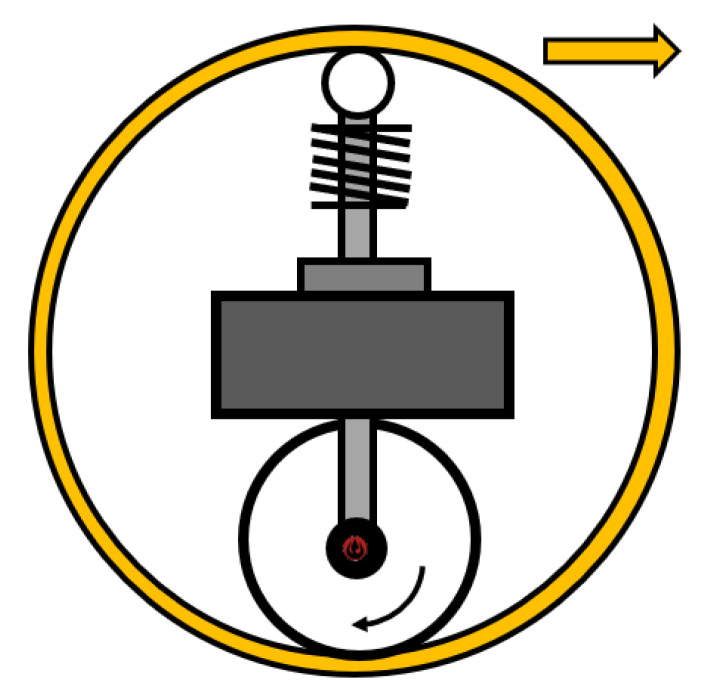
Principle sketch of a single wheel mechanism.

A special mechanism (Figure 1) is used to control the SR with a single wheel. A. Halme et al. gave the first description of this method in 1996 at the University of Helsinki. The robot has one active wheel, being located inside the shell, along with an internal drive unit (IDU), together with a spring-loaded or fixed mechanism, ensuring constant contact with the inner shell [29]. The advantage of this mechanism is the easily adjustable speed of the SR determined by the speed of the motor wheel. The direction of the SR is controlled by turning the wheel.

The mechanism is one of the simple and low-cost platforms, but there are also many challenges posed. The problem emerges at high speeds, where slipping between the wheel and the inner shell of the SR might occur. This issue can be reduced by adjusting the spring tension between the spring-loaded system, IDU, and the wheel. Nevertheless, the friction force gradually increases, thus resulting in higher power consumption. Another issue with being rolling down is the well balanced design of this mechanism. Today, we do not see many SRs with this type of mechanism, since many of the issues mentioned above must be resolved [30].

Later, in 2011, Q. Zhan described a project [31] having been based on the concept of a robot [29]. This team created a robot called BHQ-III, its name derived from the abbreviating Boltzmann-Hamel equation, and its creation is associated in a variety of ways with this equation. This mechanism consists of a single drive motor that is directly connected to the drive wheel. The drive wheel directly touches the inner shell of the SR, as in the previous case. Furthermore, the second motor is used to control the IDU, which directly controls the rotation of the robot. Undoubtedly, this principle ensures that the SR can move with an almost zero turning radius, accounting for a high degree of holonomy [31]. The primary advantage is that the platform touches the inner shell of the robot at 4 points, whilst one-touch is provided by the drive wheel, and the other 3 are provided by the sponge wheels, by means of which higher friction is achieved, and the robot can drive on uneven terrain.

#### 2.2.2. Hamster Ball

**Figure 2:**
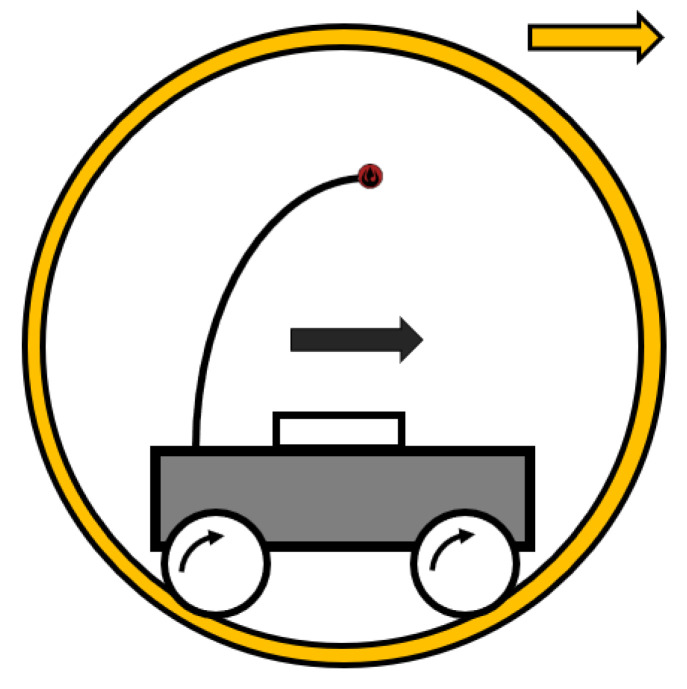
Principle sketch of a Hamster ball mechanism.

This name in itself indicates what type of mechanism is used to control the SR. In most cases, a small-wheeled robot is placed in the ball as a remote-control car (Figure 2). Based on the movement of the remote-control car, the SR also moves. The first described SR of this kind was named SPHERICLE, and its mechanism was designed by A. Bicchii [32]. The internal robot was designed as a control unit with two wheels. The advantage of this type of driving mechanism is the ability to stop on a sloping surface, and, in line with this, it can roll down from it in a controlled manner. Several research teams have designed SRs on this principle, such as References [33,34,35,36]. Conclusively, these SRs differ from the design of the internal robot.

This type of driving mechanism cannot move holonomically, and its movement more resembles driving a car. The movement characteristic might change if the internal robot “hamster” uses a differential chassis or omnidirectional wheels [30,36,37]. As far as the movement characteristic is concerned, it also changes when the internal robot will be a controlled ball [35]. Hence, the SR can change direction immediately and has holonomic properties. The main disadvantage of this mechanism is the slippage and driving on uneven surfaces. If the internal robot jumps in the air, by chance, the SR becomes uncontrollable for a short time.

### 2.3. The Gravity Driving Method

The gravity driving method is based on a change of the Center of Mass (CoM), which creates torque that, in turn, drives the robot toward the desired direction.

#### 2.3.1. Shifting Masses

**Figure 3:**
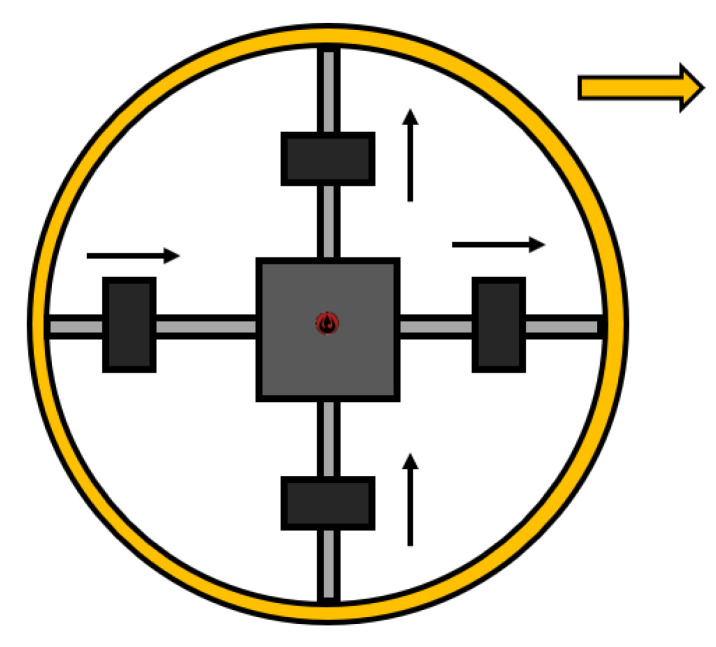
Principle sketch of a shifting masses mechanism.

R. Mukherjee et al. designed a new concept of the driving mechanism for SR called Spherobot [38]. The robot (Figure 3) disposes of a significant portion of its mass located in the geometric center of the shell. This SR requires, on the whole, minimum energy to spin the outer shell. In addition, the mechanism connects the geometric center with the inner shell of the robot using 4 shafts on which the masses are located. By moving these masses, the CoM of the robot changes, and the SR begins to roll [38].

Later on, this design was refined in References [39,40], by distributing the masses and shafts of the SR. It has the possibility, in conjunction with its design, to constantly accelerate, decelerate, and maintain a constant speed.

#### 2.3.2. Pendulum

**Figure 4:**
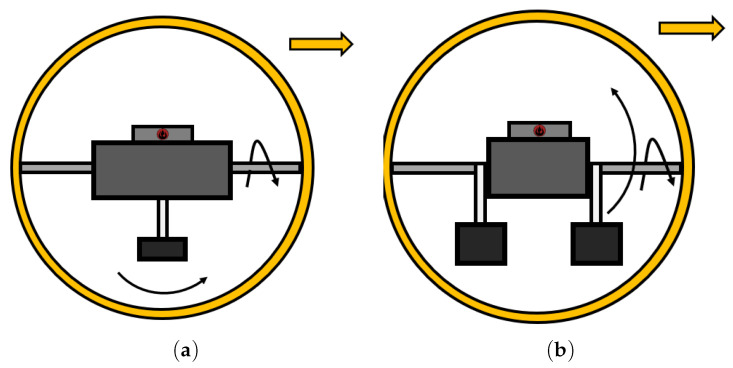
Principle sketches of a pendulum mechanism. (**a**) Single pendulum mechanism. (**b**) Double pendulum mechanism.

The mechanism based on the pendulum principle is very popular today, with several SRs coupled with the principle emerging in References [3,17,41,42,43,44,45,46,47]. The principle is based on a shaft that is directly connected to the inner shell of the SR, and a pendulum that is connected in the middle of the shaft, which rotates around the shaft (Figure 4). By changing the position of the pendulum, the CoM is shifted, and SR begins to roll. Shifting the pendulum to the sides (right, left) causes shift of the CoM; hence, the SR will start rotating in the appropriate direction. F. Michauda and his team came up with this idea first, and they designed an SR with the name Roball [48]. This robot was designed as a children’s toy. For simplicity of the model as a counterweight (pendulum), batteries were used, which are located at the bottom of the SR.

The disadvantage of this design being put forward is that the SR does not move holonomically, being manifested in a change of direction in a certain radius. There is a frail balance between the design of an SR with this principle and its internal components.

#### 2.3.3. Double Pendulum

One of the similar mechanisms used two pendulums (Figure 4). B. Zho et al. came up with this idea, thus designing an SR with an elliptical shell [49,50]. The design interesting in that the robot can rotate in place. Based on this mechanism, other SRs were designed on a similar principle in References [51,52,53,54,55]. An interesting example of the use of a pendulum is Reference [56], where 4 pendulums are used for steering the SR.

Comparing the shifting masses method with a pendulum mechanism, the main differences are as follows. The shifting mass principle is holonomic, which means that the direction of movement can change immediately. However, the controls are more complicated because the main processor must keep real-time orientation data, as well as distance data of all masses. Another disadvantage of the original design proposed in Reference [38] is the slow change of position of the mass, causing slow movement. This problem is solved in Reference [57], where the authors propose a robust higher order sliding mode controller. A second order super twisting sliding mode controller can attain the desired trajectory, as well as improve the performance of the typical non-linear robust controller. This sliding mode controller proves that the proposed controller can reduce control chattering and achieve robust operation in the presence of external disturbances and model uncertainties, as well. The authors use a super twisting algorithm to drive the error and its derivative to zero for asymptotic convergence.

#### 2.3.4. Gimbal Mechanism

**Figure 5:**
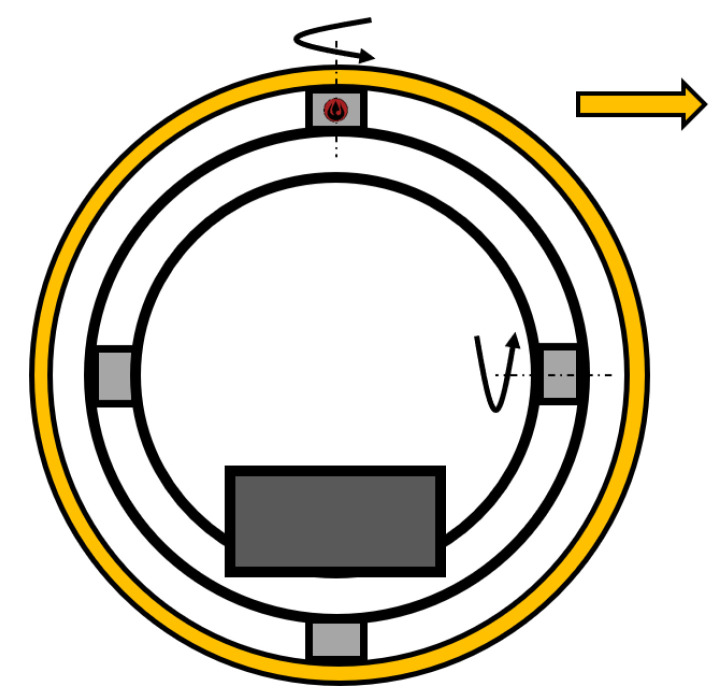
Principle sketch of a gimbal mechanism.

M. Kabał et al. designed the SR Roball in Reference [58], which was controlled by a new type of driving mechanism, i.e., the gimbal mechanism (Figure 5). Later, in 2006, a team led by Y. Ming changed the configuration of the gimbal robot and built a new SR with the name HIT [59]. The mechanism is designed in a way that the steering and driving mechanisms are independent of each other. The SR combines two motors, one for rotating the IDU in a circular path inside the shell of the SR, and the other for movement. The mechanism for moving forward/backward and the turning mechanism are self-dependent, which means HIT has non-holonomic properties. Several robots have been designed using this principle, such as BYQ-III, in Reference [60], and others, in References [53,61,62,63].

#### 2.3.5. Dynamics

The displacement of the robot’s centrum of gravity is:

(4) $$\tau =\frac{m}{M}x,$$

where *m* is the weight of the moving mass, *M* is the overall mass of the robot, and *x* is the horizontal displacement of the moving mass with regard to the robot’s geometrical centrum.

The maximal torque of the robot is:

(5) T=Mq(τ−ξ),

where *g* is the gravitational acceleration, and ξ is the rolling resistance arm.

The SR driven by CoM shifting is capable to maintain speed up to:

(6) $$v_{max}=min\left(2\pi \kappa f_{max}R,\frac{P_{max}}{Mg\left(\frac{\xi }{R}+sin\Theta \right)}\right),$$

where $f_{max}$ is the maximal speed (in rotations per second) of the driving motor, κ is the effective gear ratio between the motor and the ball (moving mass), *R* is the outer radius of the robot, $P_{max}$ is the maximal constant output power of the motor, and Θ is the slope of the terrain.

The maximal slope of the terrain on which the CoM-shifting SR may stand still is:

(7) $$\Theta_{max}=min\left(\arcsin\left(\frac{\tau_{max}}{R}\right),\arctan\left(\mu \right)\right),$$

where μ is the static friction coefficient between the SR surface and the terrain surface, and $\tau_{max}$ is the maximal displacement of the robot’s CoM. If we assume the inner structure of the directly-driven SR (see Section 2.2) does not rotate inside the robot, the same formulas are valid as in the case of CoM-shifting.

### 2.4. The Angular Momentum Driving Method

Another way used to control SRs is using the control moment gyroscopes (CMG). By spinning the flywheel and then rotating it around the axis of the robot, one might control the SR based on maintaining the angular momentum. As the CMG angular velocity increases, the torque also increases. A unique feature of using a CMG is that these systems have reaction forces in all three spatial dimensions. Specifically, if a CMG is spinning around the X-axis and is rotated about the Y-axis, then there will be a torque around the Z-axis. Many robots have been designed on this principle, such as in References [23,64,65,66].

**Figure 6:**
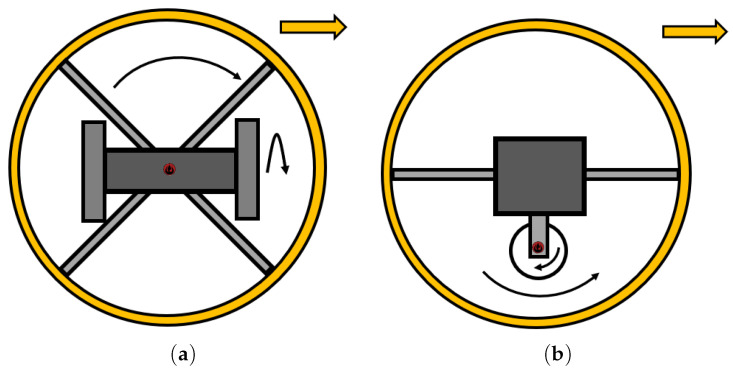
Principle sketches of an angular momentum driving method. (**a**) Mechanism of orthogonally mounted motors. (**b**) Flywheel mechanism.

S. Bhattacharya et al. designed the first SR which used angular momentum to move [67]. The SR includes two pairs of orthogonally mounted motors (Figure 6), which are connected to the shell. As a result, the shell rotates in exactly the opposite direction to that of the motor. The individual motor spins with respect to the vertical and horizontal axes of the ground, and in this way, it is possible to control the movement and direction of the SR. The advantage of this mechanism is that the SR can move holonomically. SRs designed based on similar principles are proposed in References [68,69,70].

The SR with one flywheel was designed by G. Shu [71]. The SR was designed on the principle of a pendulum, but, instead of a mass, a flywheel was placed on the pendulum (Figure 6). An acceleration or deceleration of the CMG causes the SR to start rotating. In special conditions, an SR with this type of mechanism can have a holonomy property. In 2009, an SR with one flywheel was designed on a similar principle in Reference [71]. This SR was designed by Q. Jan et al. and named BHQ-5 [72]. The rationale behind it was to use a flywheel on the pendulum. With the CMG placed where the mass of the normal pendulum system would be, the SR can rotate itself, depending on how the CMG rotates. Furthermore, depending on the orientation of the CMG and how it is moved, it can also increase the angular momentum of the SR, as well, providing more torque than that which would be provided by just a normal pendulum and mass.

J. Chen et al. designed an SR with two flywheels, in Reference [73], on a similar principle as that presented in Reference [71]. This means that the SR was able to overcome much sharper slopes than all other SRs and, at the same time, had high stability.

#### Dynamics

The torque of the robot with regard to surface is:

(8) $$T=min\left(J\frac{d\omega }{dt},\mu MgR\cos\Theta \right),$$

where *J* is the momentum of inertia of the flywheel, and $\frac{d\omega }{dt}$ is its angular velocity rate. If a very powerful motor is used to drive the flywheel, the only limit of the robot’s torque (hence its acceleration) is the friction between the ball surface and the surface of the terrain. It allows for achievement of lower response time of the robot’s motion control. The dynamics of the robot can be simplified into the following equation:

(9) $$\left(MR^{2}+J_{R}\right)\frac{dv}{dt}=T-Mg\xi ,$$

where $J_{R}$ is the momentum of inertia of the robot, excluding the flywheel. If we assume the maximal speed is achieved when maximal torque is applied, the maximal short-time speed of the robot on a horizontal surface is:

(10) $$v_{max}=\frac{dv}{dt}{|}_{T_{max}}\cdot \frac{\omega_{max}}{\frac{\mu MgR}{J}}=\frac{J\omega_{max}\left(\mu R-\xi \right)}{\mu (MR^{2}+J_{R})},$$

where $\omega_{max}$ is the maximal angular velocity of the flywheel. On the other hand, the robot is not capable to maintain constant speed in the case of constant rolling resistance because it would require infinite speed of the flywheel. The maximal time the momentum-driven SR is capable of staying still on a sloped surface is:

(11) $$t_{hold}=\frac{\omega_{max}}{\frac{d\omega }{dt}}=\frac{J\omega_{max}}{Mg\cdot max(R\sin\Theta ,0)}.$$

If the rolling resistance arm ξ exceeds the term R·sinΘ, the robot is capable of staying still on the a small slope without actually rotating the flywheel.

### 2.5. Some Other Types of Driving Mechanisms

**Figure 7:**
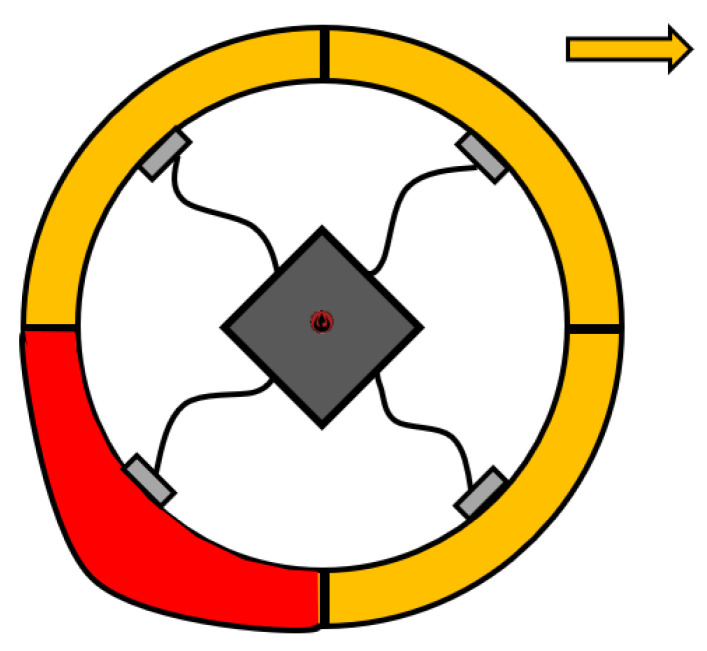
Principle sketch of deforming the outer shell.

SR can also be controlled by a change of the surface structure. M. Srtus et al. designed an SR, which moved by deforming the outer shell [74]. The outer shell consisted of 4 sections of dielectric elastomeric sections, which can be transformed by an electric field. Transformation of the sections in sequence will cause the robot to roll. Later, K. Wait et al. designed an SR, presented in Reference [75], which improved the idea of Reference [74]. The robot closely resembled a football and moved by inflating and deflating individual air segments (Figure 7). Depending on which segments are filled with air, it is possible to move the ball on an accurate path. This type of system can provide holonomic motions.

**Figure 8:**
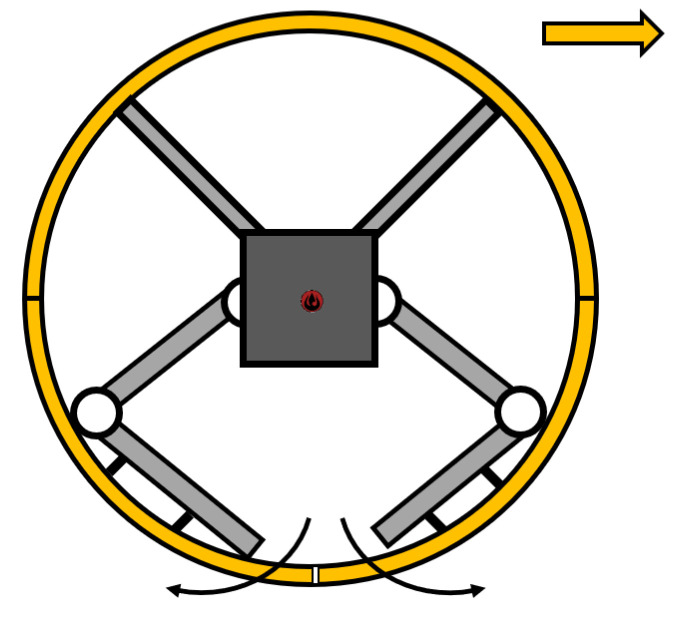
Principle sketch of a hybrid SR.

Another possibility for the SR to move is a hybrid mechanism that allows the SR to roll or walk. One of the designs was performed by S. Mahboubi, who designed an SR with four legs [76]. This design was improved by T. Aoki, who designed a fully hybrid SR [77] called QRoSS, originally designed as a reconnaissance robot (Figure 8). It has the possibility of rolling on a flat surface, and, in the case of an uneven surface or an obstacle, it will extend its legs, and the robot will overcome the given unevenness. A similar proportion of hybrid robots can operate on a similar principle, such as in References [78,79,80].

#### Dynamics

**Figure 9:**
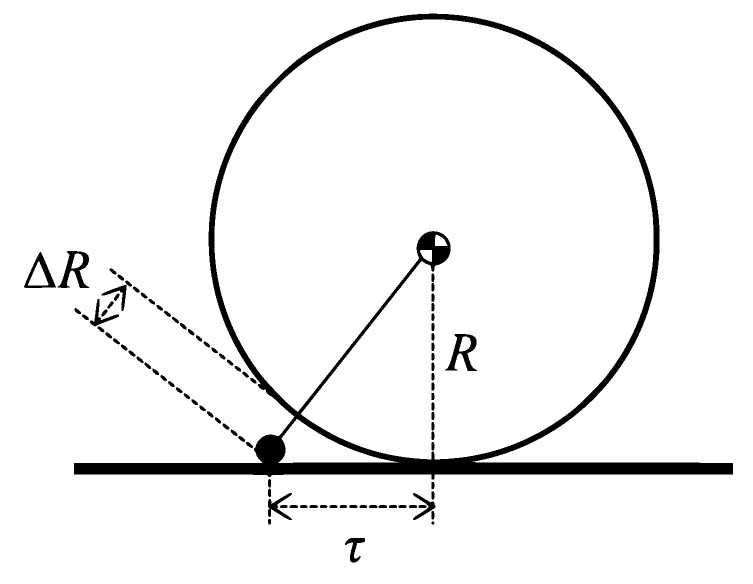
Shift of the contact point.

Deformation of the outer shell basically shifts the point of contact between the surface of an SR and the terrain. Such a shift has the same effect as CoM-shifting. We may estimate the shift of the contact point as shown in Figure 9, assuming the centrum of gravity will stay in the center of the robot:

(12) $$\tau =\sqrt{{\left(R+\Delta R\right)}^{2}-R^{2}}=\sqrt{2R\Delta R+\Delta R^{2}},$$

where ΔR is the local increase of the outer shell radius caused by deformation. The deformation allows greater τ than mass-shifting, e.g., when $\Delta R>R(1-\sqrt{2})$, the contact point is outside the radius of the robot.

### 2.6. Comparison of Each Mechanism

Each of the methods has both pros and cons. When we focus on the driving characteristic of the direct driving method, the torque of the motor is transmitted directly to the robot shell, as mentioned above. This force is comparable to the gravity method with gravity focus. The forces can be increased, which makes it possible for the SR to increase the speed of movement and, thus, overcome obstacles on the part of the robot. The angular momentum driving method uses a large kinetic force. Because a balance of momentum is needed, control is quite difficult; therefore, it is necessary to plan the trajectory of the robot.

Considering the driving capability, one of the most important characteristics is omnidirectional mobility since it is a unique feature of an SR, as described in the first paragraph. Omnidirectional mobility (holonomy), in particular, according to its definition, indicates that the robot can move in any direction at any moment, using degrees of freedom (DoF) for movement.

An SR that uses the gravity driving method can move holonomically, but trajectory planning is more difficult. The reason is that the orientation and movement of the SR are based on a continuous change of CoM using the masses. Additionally, the construction of such an SR is fairly demanding. A simple definition of holonomy is the ability to make sharp turns at any time.

However, the power of an SR with these two methods is limited because the CoM cannot be moved outside of the shell.

SRs with flywheels are not able to achieve omnidirectional holonomies because the direction of the momentum changes gradually, for which gradual change of direction is obvious.

Table 1 shows a brief comparison of some types of driving mechanisms of SRs.

**D** indicates the direct driving method, **G** indicates the gravity driving method, and **M** indicates the angular momentum method. **v** indicates that the robot is able to perform a specific movement, and v* or number* indicates that the robot can perform the movement in specific conditions.

Table 2 shows a functional feature comparison of some types of driving mechanism of SRs.

## 3. Inertial Sensors

In order to estimate the attitude and direction (3-DoF rotation) of the spherical robot, inertial sensors need to be installed onboard the robot. The rotation of the robot can be expressed by several structures, including:

- **Quaternion:**
  (13) $$q=\left(w\;x\;y\;z\right)=\left(cos\vartheta \;n_{x}sin\vartheta \;n_{y}sin\vartheta \;n_{z}sin\vartheta \right),$$
  where $\left(n_{x},n_{y},n_{z}\right)$ are the coordinates of unit vector expressing arbitrary axis of rotation and is the angle of rotation around that axis.
- **Rotation matrix T:** with dimensions 3×3, which defines the transformation between the robot’s local coordinate system and the global coordinate system, or vice versa.
- **Euler angles**: a sequence of 3 rotations around fundamental axes of the robot’s coordinate system, e.g., Z-Y-X, named yaw Ψ, pitch Θ, and roll φ.

The rotation of the robot can be measured by a gyroscope. There are 4 working principles:

- **Mechanical rotating gyroscope**: may directly measure the attitude of the robot. The oldest type, widely used in aircraft and missile control systems. It is based on the principle that the attitude of a flywheel in a gimbal is maintained regardless of the orientation of the robot or aircraft itself. The main disadvantages are larger dimensions and mass, higher power consumption needed to rotate the flywheel, and higher price. Hence, it can be used only on large robots [81].
- **Ring laser gyroscope (RLG)**: estimates the angular velocity of the robot’s rotation around one axis and is based on the Sagnac effect. It measures the phase shift between two beams of light, traveling in opposite directions in loop formed by multiple mirrors. The phase shift is proportional to the area of the loop A and the angular velocity of the rotation perpendicular to the area:
  (14) $$\Delta \Phi \approx \frac{8\pi }{\lambda c}\left(\omega \cdot A\right),$$
  where *c* is the speed of light, and λ is its wavelength. RLGs are very precise but have large dimensions in order to keep the area of the loop as large as possible [82].
- **Fiber optic gyroscope (FOG)**: works on the same principle as RLG but uses an optic fiber to guide the light around the loop. The fiber can be coiled into multiple turns, which multiplies the effective area of the loop, thus increasing the sensitivity of the sensor, while keeping the dimensions small. These gyroscopes are replacing the mechanical ones in both aviation and military technologies. The main disadvantage of FOG is the high price, rendering it costly to deploy in small, single-use robots [83,84].
- **MEMS vibrating structure gyroscope**: estimates the angular velocity of the robot’s rotation around single axis by measuring the Corriolis force affecting a vibrating mass. The main advantages are the small dimensions, low power consumption, and low cost, thanks to the MEMS (Micro Electro-Mechanical System) technology, which allows their usage in small, low-cost robots and mobile devices. MEMS gyroscopes have significantly lower precision than RLGs and FOGs [85].

If an FOG, RLG, or MEMS gyroscope is used in a spherical robot, three independent axes of rotation have to be measured. Many manufacturers provide 3 sensors oriented perpendicular to each other in single sensor module. The readings of the angular velocity need to be integrated to obtain the absolute rotation (expressed as quaternion, rotation matrix, or Euler angles). It results in increasing error of the estimated rotation [86]. This gradually increasing error can be compensated toward some constant level by using accelerometer and magnetometer to obtain the absolute Euler angles with respect to the horizontal: North orientation.

The accelerometer measures the sum of the linear acceleration of the object and gravity acceleration:

(15) $$a_{acc}^{\prime }=a_{robot}^{\prime }+g^{\prime }.$$

The acceleration of the spherical robot can usually be neglected with respect to the gravity acceleration. The roll and pitch angles (Z-Y-X Euler convention) can be computed from acceleration as:

(16) $$\Phi_{acc}=atan2\left(-a_{y}^{\prime },-a_{z}^{\prime }\right),$$

(17) $$\Theta_{acc}=atan2(a_{x}^{\prime },\sqrt{{a_{y}^{\prime }}^{2}+{a_{z}^{\prime }}^{2}}.$$

There are 3 main types of accelerometers:

- **Piezoresistive accelerometers**: are simple, low-cost and DC-responsive devices that can measure constant acceleration, such as gravity. The major drawbacks of piezoresistive sensing are the temperature-sensitive drift and the lower level of the output signals. The sensing element consists of a cantilever beam, and its proof mass is formed by bulk-micro-machining. The motion of the proof mass due to acceleration can be detected by piezoresistors in the cantilever beam and proof mass. The piezoresistors are arranged as a Wheatstone bridge to produce a voltage proportional to the applied acceleration [87,88].
- **Piezoelectric accelerometers**: do not respond to the constant component of accelerations. In a piezoelectric accelerometer, the sensing element bends due to applied acceleration, which causes a displacement of the seismic mass, and results in an output voltage proportional to the applied acceleration [89].
- **Differential capacitive accelerometers**: have low power consumption, large output level, and fast response to motions. The displacement of the proof mass can be measured capacitively. In a capacitive sensing mechanism, the seismic mass is encapsulated between two electrodes. The differential capacitance is proportional to the deflection of the seismic mass between the two electrodes. Better sensitivity is also achieved due to the low noise level of capacitive detection. Differential capacitive accelerometers also have DC response. Currently, this kind of accelerometer has widely been used in most applications, especially in mobile and portable systems and consumer electronics [90,91].

The 3-axis magnetometer (magnetic compass) allows for estimation of the direction of the robot from the measured magnetic induction vector

(18) $$\psi_{mag}=atan2\left(-B_{y},B_{x}\right),$$

where $B_{x}$ and $B_{y}$ are the components of the magnetic induction vector in global coordinates, which can be computed from measured vector $\left(B_{x}^{\prime },B_{y}^{\prime },B_{z}^{\prime }\right)$:

(19) $$B_{x}=B_{x}^{\prime }cos\Theta +B_{y}^{\prime }sin\Phi sin\Theta +B_{z}^{\prime }cos\Phi sin\Theta ,$$

(20) $$B_{y}=B_{y}^{\prime }cos\Phi -B_{z}^{\prime }sin\Phi .$$

So far, different types of magnetic field sensors, such as Hall sensors, semiconducting magnetoresistors, ferromagnetic magnetoresistors, fluxgate sensors, superconducting quantum interference device (SQUID), resonant sensors, inductive magnetometers, etc., have been developed for various applications. In the following sections, these types of magnetic field sensors are discussed:

- **Hall sensors**: are based on the Hall effects. These sensors alter its output voltage in relation with the magnetic field. More than 90% of all magnetic field sensors are Hall sensors. Hall sensors are used in proximity switching, positioning, speed detection, and current sensing applications [92,93].
- **Semiconductor magnetoresistors**: change their electrical resistance in response of external magnetic field. Though semiconductor magnetoresistors are less common than Hall sensors. Modern semiconductor magnetoresistors are fabricated as a serial connection of many miniature elements on one chip. The main disadvantage of these sensors is their quadratic characteristics, which does not allow their use in small fields. Both magnetoresistors and Hall sensors are sensitive to the magnetic field perpendicular to the surface [94].
- **Ferromagnetic Magnetoresistors**: can be classified as anisotropic magnetoresistance (AMR)-, giant magnetoresistance (GMR)-, and spin-dependent tunneling (SDT)-based sensors [95].
- **Fluxgate sensors**: are classical precise sensors developed in the 1930s. They can measure DC and low-frequency AC fields. Fluxgates are expensive, bulky, and power-consuming devices [96,97].
- **Superconducting Quantum Interference Device (SQUID)**: are based on superconducting Josephson junction and flux antenna. These extremely sensitive devices measure magnetic field changes, rather than absolute field value. SQUIDs today are used for biomagnetic experiments and for measurement of magnetic properties of samples that are small or magnetically very weak [98].
- **Resonant magnetometers**: measure the total field value (scalar), rather than its vector. This means that the reading is not dependent on the field direction (with the exception of possible dead zones). This may be an advantage in cases when the directional information is not required, or it can be derived using other sensors. In such cases, the field measurement is easy, as the direction of the sensor head is arbitrary. The main advantage of resonant magnetometers is that they are absolute instruments with very small uncertainty. The disadvantage of resonant magnetometers is that they usually have limited field range, and they fail at small fields [99,100].
- **Induction magnetometers**: are based on the Faraday induction law, which means that the voltage sensitivity is proportional to the frequency [101,102].

There are multiple sensor fusion techniques for a gyroscope + accelerometer + magnetometer system, which is usually a variation of extended Kalman filter or complementary filter [86].

## 4. Sensor Equipment

As we mentioned in Section 1, the following sensors can extend the possibilities of the SR. The sensors were specified in Reference [1], and the same sensors were specially designed for the intended purpose of reconnaissance, during emergencies in road tunnels or during a traffic accident.

We will deal with cameras in the visible spectrum and IR spectrum. These cameras can be used for visual monitoring space around the robot. In addition, we will deal with gas and temperature sensors. These sensors can determine the state of the environment. Lastly, the described sensor will be LiDAR, which is appropriate for scanning the environment and making a 2D map, as well as might be used for precise obstacle detection.

### 4.1. Cameras (Smallest and Low-Cost)

The main topic is to determine the smallest possible camera with sufficient resolution with the most acceptable cost for use in our application.

The image capture device may be a CCD-based camera, or a CMOS-based one. In principle, CMOS devices are smaller and cheaper than CCD cameras, but the quality of CMOS images is not as good as with a CCD camera. In general, better image resolution means a larger image size and higher processing and transmission performance. The trade-off between the quality of visual data and the consumption of system resources depends on the requirements of the application. For applications that require higher image quality (e.g., face recognition as part of security surveillance), a CCD camera is a better choice. For applications that require to capture a large number of images do not require high resolution (such as tracking objects in the wild), CMOS is a better choice. In addition to the requirements of the application, the chosen device depends on the trade-off between price, size, measuring spectrum, energy consumption, and the resolution of the visual data.

Our research focused primarily on CMOS cameras, due to the fact that it is not necessary to use high-resolution images, and the price is lower than CCD cameras.

#### 4.1.1. Cameras Operating in the Visible Spectrum

A large number of mini cameras operate in the visible spectrum. In Reference [103], the OV9655 scanner was used as a barcode scanner, and, in Reference [104], the scanner in a wireless network was used, due to its low energy consumption.

W. Song et al. developed a survey robot presented in Reference [105] that is used to inspect cable tunnels. They used the OV7670 CMOS sensor for this application. The sensor was chosen for its small size and low power consumption. However, with the exception of the ideal conditions, the design for real conditions would still require further adjustments. Another interesting application was presented in Reference [106], where gestures were sensed using the mentioned camera sensor and subsequently recognized by the FPGA. Alternatives are proposed in other implementations in References [107,108].

An interesting area of use of mini cameras is the creation of wireless networks to capture a certain area. W. Qi et al. created an intelligent home video surveillance presented in Reference [109], where they designed a wireless monitoring system. In this case, they used the OV2640 camera. Mini cameras are also quite frequently used in healthcare, namely in endoscopy and laparoscopy, where the effort is to provide the smallest possible tools for intervention in the human body. Thus, Y. Zhang et al. created a capsule endoscope [110]. This endoscope uses wireless image transmission, where the aforementioned camera element was used.

Another frequently used camera is named MT9M001. In 2011, it was used in high-speed image processing [111]. A team led by L. Qin created a system for high-speed image detection and processing. This system is typically used in photoelectric tracking or communication with a space laser. In the field of biomedicine, this sensor has been used as part of an optical demographic system [105]. The camera was used to increase the accuracy of particle size detection. Alternatively, in Reference [112], it was a component in X-ray spectroscopy.

#### 4.1.2. IR Cameras

Infrared radiation is often confused with thermal radiation (according to the sensitivity of the human body), but the fact is that the surfaces of bodies emit almost all types of electromagnetic radiation. However, it is true that objects at room temperature emit the most radiation in the IR band 8–12 µm. From about 700 °C, heat radiation, at wavelengths, is visible to the human eye. Colors range from “dark glow” to dark red, red, and yellow to white.

A. Gecza et al. created a car passenger detector based on an infrared camera [113]. They used a small and low-cost infrared camera called AMG8833 for this experiment. The position of the sensor, the resolution, the ambient temperature, and many aspects were taken into account, as the camera has certain limitations. The camera was validated in a laboratory environment at room temperature, where it was possible to distinguish a person from the background. Four basic positions were considered in the car, with people being able to sit in the front or rear seats, or more (0–5 people) at the same time. The result of the work was to point out that it is possible to detect passengers in the vehicle even with a cheap solution. In addition, in 2020, during the COVID-19 pandemic culmination, non-contact temperature measurement became more and more common. V. Ionescu et al. created an overview of low-cost thermal sensors, where, in Reference [114], they also mention the aforementioned camera. Alternatively, this type of sensor has been reported in patient monitoring that respects patient privacy [115,116].

Another area of using infrared cameras is in the design of an intelligent air cooler system [117]. P. Du designed this proposal, together with his team, where they used an infrared camera MLX90640. Using an infrared camera, they detect an area of uneven heat dissipation and, in turn, create a temperature grid based on the image. At the same time, the camera was also used for temperature calculation methods for electrical equipment [118]. They point out a demand for monitoring the temperature of electrical machines because, when the temperature rises too high, it is likely to cause serious accidents.

#### 4.1.3. Comparison of Camera Sensors

The following, Table 3, summarizes all used sensors, together with their basic properties.

### 4.2. Gas Sensors (Smallest and Low-Cost)

The main idea is to establish which are the smallest possible gas sensors with sufficient accuracy and low cost.

In enclosed spaces with little possibility of ventilation, or during a traffic accident in the tunnel, it is inevitable to know the concentration of dangerous gases in the air. In conjunction with this, prolonged exposure to a hazardous gas in a closed room can result in permanent damage to human health or even death. Therefore, the robot will read the values of CO${}_{2}$ (Carbon dioxide) and CO (carbon monoxide) concentrations. Table 4, below, shows the given concentration intervals of individual gases and their consequences [42,126].

When measuring gases concentration, such as carbon dioxide, oxygen, or methane, the term concentration is used to describe the amount of gas by volume in air. The two most common units of measurement are parts per million and percent concentration. Parts per million (ppm) is the ratio of one gas to another. For example, 1000 ppm of CO means that, if we could count a million molecules of gas, 1000 of them would be from carbon monoxide, and 999.000 molecules would be some other gas.

#### 4.2.1. CO${}_{2}$ Sensors

Domestic air monitoring stations, measuring mainly carbon dioxide, are largely made from cheap gas detectors. From Table 4, we can see that, if there is good air quality in the room, people can then be fully focused on their chores. In Reference [127], a dangerous gas sensor was designed using the MQ-135 sensor [128,129], which also included other dangerous gases. Alternatively, in Reference [130], the author created a simple detector of CO${}_{2}$ and CO based on the aforementioned sensor and displayed the resulting value on a display in ppm. When exceeding the limits, the system triggers an alarm in the form of a buzzer noise.

A similar work was created in the LabVIEW platform. In Reference [131], in 2020, the author created a home alarm, which also contained a CO${}_{2}$ sensor, MQ-135. The system can monitor the following parameters: temperature, gas concentration, and the presence of smoke due to fire, as well as an audible warning when exceeding normal values. Reference [132]’s work was created in the given tool, which uses a sensor of the MG-811 series for CO${}_{2}$ sensing [133]. Online solutions for monitoring environmental parameters using Internet of Things (IoT) techniques help to collect values of parameters, such as pH, temperature, humidity, and CO${}_{2}$ concentration. The use of sensors allows intensive control of environmental pollution caused by industrial manufacturing. Reference [132] deals with the control of pollution caused by the untreated disposal of waste. In the measurement of air pollution in Reference [134], the author uses the sensor MQ-135 for sensing ambient gases around the landfill. The scholar designed a platform based on gas sensors to monitor the surrounding gases from solid waste repositories using a given mobile phone sensor. A similar work with the same sensor was focused on sanitary landfills [135]. The authors dealt with two parameters for this type of landfill. Groundwater quality and greenhouse gas concentrations were measured simultaneously. The data from the sensors are sent to the cloud, where they are processed. If the limit value is exceeded, an SMS will be sent.

Recently, A. Gecza et al. created a prototype for measuring air quality [136]. They created a low-cost prototype for air quality monitoring that is small in size, easy to use, and compatible with a smartphone. The prototype was tested on the streets of Budapest. Alternatively, there is the possibility of using the facility in connection with the COVID-19 pandemic. In relation to air quality in hospitals, a proposal for a low-cost air quality monitor has been developed [137]. Table 4 illustrates reduced air quality results in headaches, fatigue, and lack of concentration. The hospital also works with a number of chemicals that can degrade air quality. At the same time, with a large number of people in a small space, therefore, it is necessary to constantly monitor the air quality. Reference [137] introduces a cheap variant of a monitoring device with a CCS811 sensor [138]. Alternatively, there are similar papers about air quality measurement; for example, see References [129,139,140].

In 2020, a mobile robot was created by M. Evit et al. to monitor a volcano [141]. The mobile robot was designed in Indonesia, as they are in the Pacific ring of fire and have a total of 129 active volcanoes. If an eruption occurs on a volcano, the current fixed monitoring system is not completely reliable. On the other hand, monitoring of other volcano activities is critical in this situation. Therefore, there is a need for a volcano monitoring system that can move freely and operate safely. When creating a mobile robot, we used the MG811 CO${}_{2}$ sensor, as well as a number of other sensors.

#### 4.2.2. CO Sensors

The measurement of CO is largely performed in the determination of air quality with the help of AQI (Air Quality Index). Thus, a large amount of work deals with the creation of a low-cost platform for measuring AQI, which also includes the measurement of CO concentration in the air [129,142]. Sensors, such as MQ-7 [143] or MQ-9 [144], are used for these measurements.

MS. Rane et al. created a wireless sensor network to measure AQI in real-time [145]. They focused on the cheap, affordable sensors mentioned above. The result of the work was the calculation of AQI in the main node using smaller wireless AQI sensors. Alternatively, K.M. Ng et al. introduced remote monitoring of AQI using MyRio-LabVIEW [146]. They presented a project where they wirelessly measured the value of CO concentration using the MQ-7 sensor and subsequently processed the obtained data using a platform from LabVIEW.

In the field of IoT, R. Firdaus et al. created an air quality monitoring system using LPWAN LoRa [147]. They created a system that uses IoT technology to monitor temperature, pressure, humidity, and the concentration of CO and CO${}_{2}$. The aforementioned MQ-7 sensor was used to measure the CO concentration. The measured data are stored in a cloud, from where they can be viewed using the Android system. Similarly, R.K. Kodali used IoT technology to measure air quality using the MQTT protocol [148]. The paper focuses on measuring dust and CO in smoke produced by factories in India. These factories are the primary contributors to air pollution as solid particles cause human health problems, such as asthma and other respiratory diseases. In the work, the author obtains values from sensors and, later on, sends them to the cloud. This measured data is displayed on the website and the mobile application. Another application of CO measurement in IoT can be found in References [149,150,151].

An interesting application of CO measurement is proposed for mining tunnels. M.M. Tao developed an alarm system based on Single-chip, which measures the concentration of ammonia and CO in tunnels [152]. When the limit values are exceeded, an alarm is triggered, and a buzzer makes noise.

S. Kouda et al. proposed modeling of the MQ-9 gas sensor, where the modeling is based on artificial neural networks (ANN) [153]. The sensor works in a dynamic environment and expresses the behavior model of the MQ-9 sensor. Accordingly, it takes into account non-linearity and sensitivity in gas, temperature, and humidity selectivity.

#### 4.2.3. Comparison of Gas Sensors

The following, Table 5, summarizes all used sensors, together with their basic properties.

### 4.3. Temperature Sensors (Smallest and Accurate)

Thermocouple probes are standard equipment for recording forest fire data for later analysis. Despite advances in technology, these commercially available probes are still expensive; thus, it is sometimes not possible to cover a large area with them. As these probes are used in a high-temperature environment, it is not possible to use any cheap temperature sensor. Currently, there are several projects [154,155] influencing the development of thermocouple probes for these purposes and available at reduced cost compared to commercial technologies. These papers are dealing with a K-type thermocouple.

There are thermocouples also used in coffee roasters [156]. The process of roasting coffee has a significant effect on the taste of a coffee drink. The roasting has several levels, and it is necessary to ensure the exact temperature in each individual process. Thermocouples are used at the entrance to measure quite high-temperatures in this area. A similar project [157] deals with a black pepper dryer, and a K-type thermocouple is used to measure the temperature. The quality of the pepper depends on a precise measurement.

In most cases, thermocouples are used in industrial applications to measure higher temperatures, such as to regulate the plate temperature [158]. Alternatively, an interesting example of thermocouple temperature measurement during lathe turning is proposed in Reference [159]. When cutting metals, the measurement of the temperature of the cutting tool is influenced by various factors. Because the lifespan of a cutting tool depends on the cutting temperature, it is important to predict the generation of heat in the tool by utilizing reliable techniques. A K-type thermocouple was used for this purpose.

There are a large number of other interesting papers where thermocouples are used [160,161,162,163].

In this paper, our aim is to determine the smallest possible temperature sensor with sufficient accuracy and relatively low cost.

#### Comparison

Table 6 describes the individual thermocouples [164,165] and their properties.

### 4.4. LiDAR (Smallest and Low-Cost)

When trying to choose the smallest possible LiDAR, we also pay attention to sufficient accuracy and affordability.

In recent years, light detection and ranging technology (LiDAR) has been used in countless applications. Over the years, the design of the LiDAR system has significantly improved, leading to a design with remarkably low cost, size, weight, and higher performance requirements. These systems can collect spatial information and are available in three variants: one-dimensional (1D), two-dimensional (2D), and three-dimensional (3D).

Many application-specific papers on LiDAR technologies have been published in the past, such as in References [166,167,168,169,170,171].

The primary area for the LiDAR’s application is autonomous vehicles. The detection of obstacles in autonomous vehicles is mandatory to maintain the safety of the driver and pedestrians and the navigation of the vehicle during the journey to the desired destination. Currently, in-vehicle warning systems are integrated to help the driver move safely on the road. However, autonomous vehicles must recognize the obstacles themselves and start moving safely around the detected objects. Several applications and suggestions have emerged that address the use of LiDAR for safe driving [172,173,174,175,176]. Based on the measured data from LiDAR, the algorithms generate an optimized route with adaptation to the current position of the vehicle, the location of obstacles, and the capabilities of the car. The route planning is updated in real-time, while new or moving obstacles are identified.

A similar use of LiDAR can be found in autonomous robots [177,178,179,180,181], which have the task of avoiding an obstacle or overcome a given obstacle to minimize damage to the robot. Like autonomous cars and robots, autonomous drones must detect an obstacle in real-time and avoid it. References [182,183] describe proposals for UAV (Unmanned Aerial Vehicles), which use LiDAR technology to detect obstacles.

Modern technologies can make life much easier for people with disabilities. An example is the design of a wheelchair for the disabled or the elderly [184,185]. Reference [184] uses LiDAR technology to avoid collisions with static and moving obstacles. Two additional ultrasonic transducers were used in conjunction with LiDAR to ensure greater safety.

Reference [186] suggests the use of a small LiDAR as an early warning for bikers before an approaching vehicle comes from behind. LiDAR is the part of an evaluation unit that communicates with an intelligent helmet that alerts the driver of the motorcycle in time when the car is approaching to prevent a serious accident. Similarly, J.V. Brummelen and his team designed a similar technology, but for cyclists [187]. They placed a LiDAR on the back seat, along with two ultrasonic sensors. Depending on the approaching vehicle, the algorithm generates appropriate warning signals so that the cyclist can assess the risk of a collision based on the distance, speed, and direction of the approaching vehicle.

## 5. Mobile Robots for Special Applications

Technological advancements in the past decade have given rise to the utilization of robotic technology in different fields: surveillance, reconnaissance, search, and rescue. For the purpose of minimizing the risks for rescuers, good practice speaks in favor of utilizing mobile robots in their teams. Examples of such an application are various, including: police robots, firefighter robots, and military.

### 5.1. Search and Rescue Robots

The main mission for the robots and their operators is to find victims, establish what their situation is, and then report back their findings based on a map of the building, tunnels, etc. These places of information will immediately be given to rescue teams preparing to save all victims being possibly found. Moreover, some other expectations from rescue robots pose: ability to autonomously find compromised and collapsed structures and provide shoring, find victims and ascertain their conditions, deliver sustenance, and communications to victims, and lastly, emplace sensors, such as acoustic, thermal, gas, etc.

The prime example of such a robot is a PackBot [188]. The PackBot is a multi-mission tactical mobile robot developed and manufactured by iRobot. Bearing in mind, it is designed to carry out dangerous missions. The robot carries out surveillance and reconnaissance, detection, building and route clearance, explosive ordinance disposal, and vehicle inspections. In line with this, it can be easily configured based on the operators’ mission needs. The PackBot features a modular design, incorporating an array of various sensors [188].

The robot has a manipulator to inspect hazardous materials. More precisely, the front bay is mounted with a wide-angle drive camera. Besides, the machine is also fitted with a small arm manipulator featuring a color camera for conducting manipulation and inspection. Last of all, the PackBot was designed with a built in thermal camera, an explosives detection sensor, and a HazMat kit to detect chemical and biological materials.

Our attention deserves a simpler version of the aforementioned robot, as well [189]. Accordingly, the authors designed a crawler rescue robot with a robotic arm attached to it. The arm handles detonation of explosives, mines, and other objects. Besides, the robot also includes sensory equipment, where vital usage of low-cost sensors, e.g., gas sensors, thermistors, humidity sensors, etc., found its application. Authors used the ZigBee communication protocol for communication, and it was controlled by virtue of an Arduino and Raspberry Pi module.

### 5.2. Fire Robots

The most popular firefighting robots in the field today were designed by Howe & Howe. The robots names are Thermite RS1-T3 and RS2-T2 [190]. Both are powered by diesel engines, controlled remotely, and can climb slopes up to 70 degrees [190]. They can blast out up to 9500 L of water and foam per minute, and, for this reason, the workhorses are intentionally designed to tackle major industrial fires, such as oil refinery blazes.

A robot named Colossus [191] saved the French Notre-Dame Cathedral. This robot resembles WALL-E and has a capacity of 2500 L of water per minute. Aside from extinguishing fire, the joystick-controlled Colossus can also haul firefighting equipment, as well, and transport wounded victims. This robot was designed by Shark Robotics company.

### 5.3. Surveillance and Reconnaissance Robots

The first interesting device for telemonitoring and surveillance is made up in a form of the Recon Scout robot [192]. It is a two-wheel mobile robot with a titanium body and wheels of urethane plastic. Forward movement facilitates the so-called tail being simultaneously the support of the robot. The robot can travel speed of 1.1 km/h. The range inside a building is 30 m, and outside is 70 m. The robot is equipped with a black and white camera.

The second robot being put forward is EyeDrive [193]. This is a four-wheeled robot produced in Israel. The system of cameras makes it feasible to obtain a panoramic view with an image definition of 2500 × 570 pixels. For the sake of sound transmission, the build in microphone enables hearing sound from a distance of 10 m. The robot’s range inside the building is 70 m, whilst it is 300 m outside. The robot can bear additional loads, such as sensors, explosives, etc.

The mobile robot SpyBowl [194] must be thrown or rolled toward the target. The outer shell, with respect of its purpose, is made from an aluminum, covered by rubber, coating in the form of a ball. In terms of audiovisual perception, it is equipped with four cameras, allowing the acquisition of static images, and with microphones transmitting the sound. Furthermore, the SpyBowl can rotate about its vertical axis, which allows us to watch all environments dynamically. Additionally, the image can be seen from each camera independently. The range of the radio transmission varies between 20–30 m inside a building, and 100–300 m outside of it.

A similar robot, regarding design, is Eye Ball [195]. It is designed for throwing, rolling, or dropping. In terms of providing audio and video transmissions in real-time, the device is equipped with one camera, providing a good quality picture. To collect complete information about the environment, Eye Ball rotates about its own axis. Its extra software makes it feasible to acquire a panoramic view. Besides, the device has IR, and, thanks to it, the camera can see in darkness. Radio and video transmission effectively work up to a distance of up to 125 m, depending on the surrounding environment.

## 6. Design of the Reconnaissance Robot

Given all the possibilities where SR can be used (Section 1), the writers of this paper identified a narrow area in which there is an effort to design their specific SR. This area covers rescues or reconnaissance applications. Thus, our idea is to design the SR that will serve as a reconnaissance SR with sensory equipment that makes it likely to provide a certain type of intervention (especially intervention in the road tunnel), so that the lives of firefighters would not be endangered during the intervention of the dangerous area.

**Figure 10:**
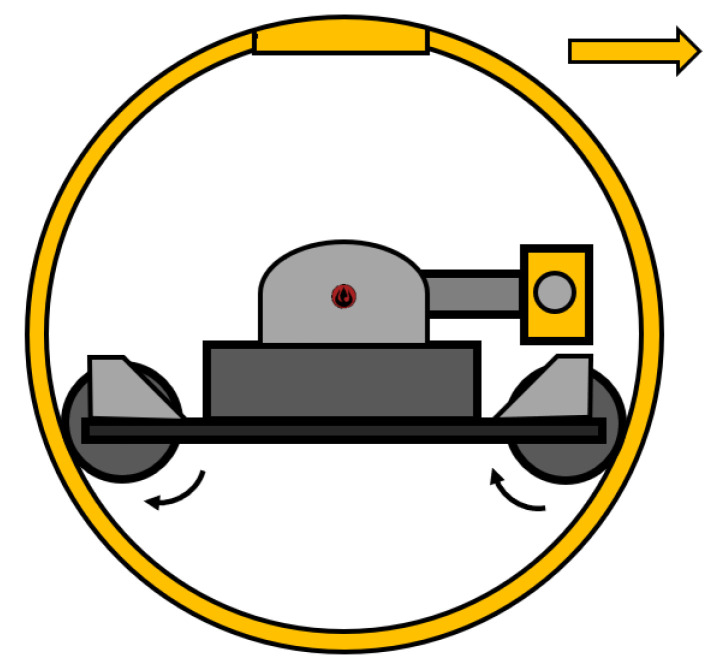
Principle sketch of our SR.

**Figure 11:**
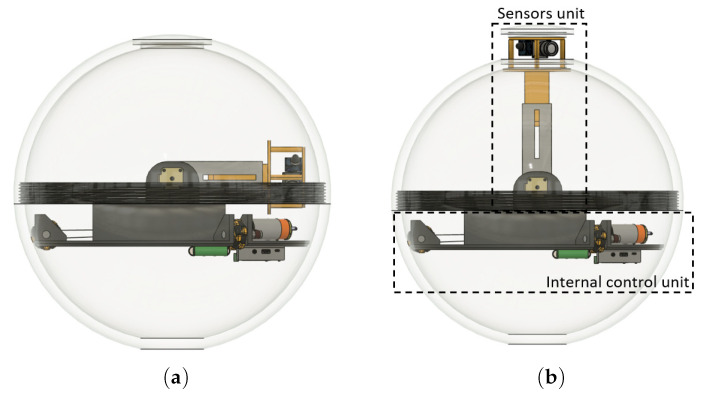
Modes of our SR. (**a**) Rolling mode. (**b**) Measuring mode.

The principle of direct driving method provides the basis for our SR (Figure 10). Let us turn to architecture of our robot in greater detail. Firstly, the internal control unit (ICU) controls and navigates the robot to the accident area, and, secondly, the sensors unit (SU), is made up of of a measuring head, where sensors collecting data about the specific environment, and the pull-out mechanism, which extends the measuring head out of the shell are placed. The SU will be protected from external influences by the shell of the SR. The advantage of this design will be that the SU is directly connected to the ICU. Thus, the measured data will not have to be transmitted by wireless technology between the two parts. This design is unique and specific and was ultimately designed specifically for this purpose. The SR features two modes. The first one is the rolling mode (Figure 11a), where the measuring head is OFF and inside of the shell. The second one is the measuring mode (Figure 11b), where the measuring head is ON and outside of shell.

### 6.1. Internal Control Unit (ICU)

**Figure 12:**
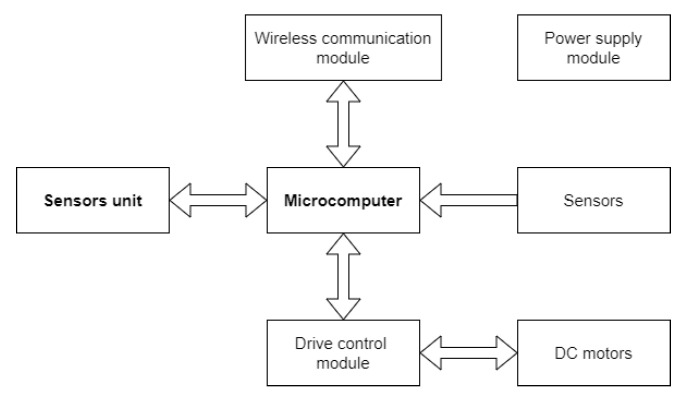
Simple diagram of an Internal Control Unit (ICU).

The foundation of the SR is the ICU (Figure 12), consisting of the following parts: control unit (microcomputer), drive control module, DC motors, sensors, power supply module, and wireless communication module.

A microcomputer with sufficient computing power was chosen as the main control unit because the control algorithm must be executed in real-time. The unit mentioned will command the drive control module, the sensors determined for robot driving, and the communication module. With respect to many previous papers, we opted for the Raspberry Pi 4 as the control unit.

The drive control module is determined to control 3 motors, driving the SR. Based on the motor’s specification given below, a module with a maximum current of 10 A was defined. In terms of the application case presented, a module with the designation MDD10A was selected.

The motors are independent of each other, with a short timing belt of two motors that will connect the omnidirectional wheels, and a longer timing belt the third motor that will connect to the omnidirectional wheel, respectively. For the usage of the omnidirectional wheels, a diameter of 80 mm appeared to be most appropriate. The reason for such an architecture is coupling of all motors and ensuring that the CoM is moved closer to the shell of the SR. In this case, DC motors 36GP-555 ABHL with a voltage of 12 V were designed. The DC motor has a ratio of 5.2, which means 1150 RPM when the motor is unloaded. A nominal torque is 3 kg·cm, at 880 RPM, with a rated current up to 2 A. When maximally loaded, the maximum current take-off is 9.5 A, based on which the drive control module was determined. An encoder is part of the motor, as well.

Sensors are required to determine the position of the ICU, movement, rotation, etc. The primary sensors of the SR taken into consideration are the accelerometer, the gyroscope, the sensors of the current flowing into the motors, and the speed sensor of the motor. These sensors will be, in most respects, used for the inertial navigation system of the robot because the SR will move in closed spaces, such as a tunnel, since it is not possible to monitor the robot’s movement there using GPS.

The power supply module is used to provide enough power for all modules in ICU and SU. This module will consist of batteries with 12 V and a step-down converter of 12 V to 5 V. In addition, a charging module will be connected to the batteries, which will be used to charge the batteries.

The last equipped module is a wireless communication module. This module will be used to receive control commands and to send measured data from each sensor and scanning device. Specifically, we can consider Bluetooth, WiFi, ZigBee, LoRa, and Thread, or using free communication bands, such as 868 MHz or 443 MHz, for the sake of wireless communication. As the SR will move in a closed space, especially in tunnels, it is necessary to bear in mind several factors play a pivotal role in the signal transmission in closed spaces and, in turn, choose the most appropriate technology.

### 6.2. Sensors Unit (SU)

**Figure 13:**
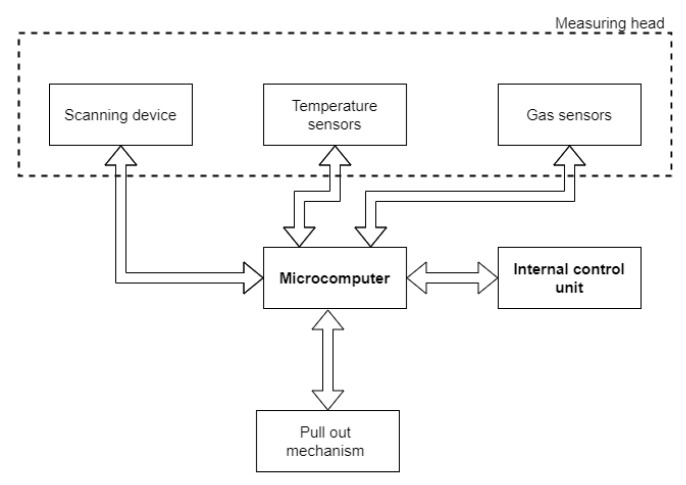
Simple diagram of Sensors Unit (SU).

The SU (Figure 13) will be used for the measurement of dangerous gases and temperature in the vicinity of the SR, and the scanning device will be used for the mapping of the space in which the SR is located. This SU consists of: a microcomputer, scanning devices (LiDAR and camera), gas sensors, temperature sensors, and pull-out mechanism.

## 7. Discussion

**Figure 14:**
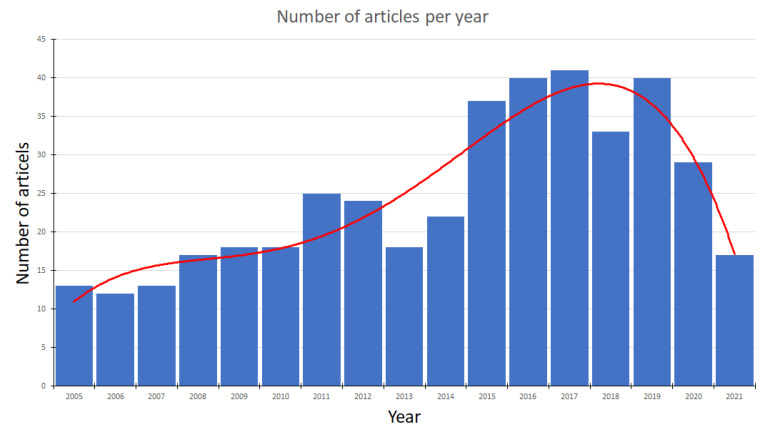
Histogram of articles about SR.

Currently, SRs are widely preferred in various fields of application, such as space exploration, monitoring, and surveillance of buildings and large areas. Despite the frequent usage of standard robots, various alternatives have also arisen, such as amphibians, hybrids in connection with a drone, or hybrids in connection with a walking robot. If we look at the histogram (Figure 14) below (based on the Web of Science), we can observe the largest expansion of SRs was from 2015 to 2019. Today, the trend with SRs is in decline (October 2021).

**Figure 15:**
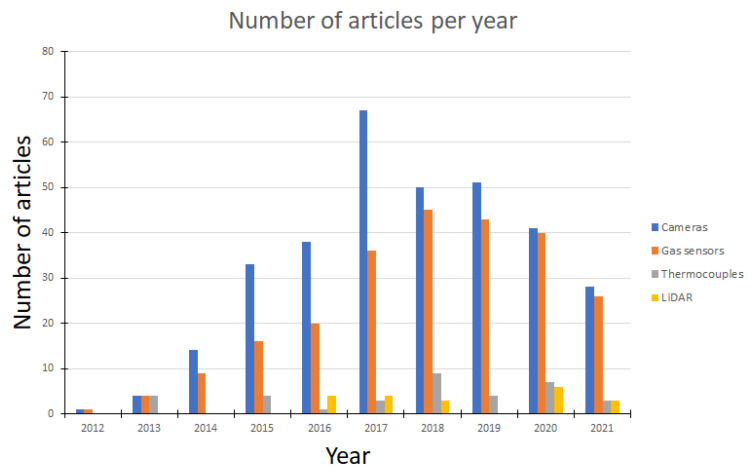
Histogram of articles about cameras, gas sensors, thermocouples, and LiDARs.

In Section 4, scholars focused on a review of articles, where sensors, such as cameras, gas sensors, thermocouples, or LiDARs, in low-cost applications, were used. In the current section, we focused on two types of cameras. First, on the account of the camera capturing the visible spectrum, we described 4 simple and low-cost cameras. We primarily identified their resolution, maximum image rate, and power consumption. The rationale for the second type of camera was its working within the IR spectrum, which turns into a description of 2 types of these cameras. We put emphasis on the same properties, such as cameras working in the visible spectrum. Histogram 2 shows the number of publications on these low-cost cameras per year. It is obvious (from histogram 2) that the trend in the use of these low-cost cameras is growing up every year. Later on, we focused on gas sensors. In general, CO and CO${}_{2}$ are the most dangerous gases for humans, so we described low-cost sensors that can measure these two types of gases. Accordingly, we focused on detection range and power consumption. Therefore, a description of the 3 most common CO${}_{2}$ sensors, as well as 2 types of CO sensors, was carried out in more detail. Figure 15 proves that the largest boom in gas detection was in the period 2017–2019. The following part of this section described thermocouples, and it included widely used thermocouple of type K. Figure 15 shows that the largest boom in thermocouples detection of temperature in low-cost design was in the period 2016–2020. The last part of this section attended low-cost LiDAR scanning. This review shows the possibility for a sensor’s usage in creating 2D map of the place. Figure 15 shows that low-cost LiDAR technology can be used very often in a simple design.

Section 5 provides insights into the various mobile robots for special applications. We focus on surveillance, reconnaissance, fire, search, and rescue robots. Many fire, search, and rescue robots use belts, and the construction is massive. On the contrary, surveillance and reconnaissance mobile robots are simple and small. These are usually four-wheeled (two-wheeled) robots or robotic balls that have environmental sensors. The perspectives in Section 5 determine, in a variety of ways, the focus of our paper.

In Section 6, we propose a new design of the SR for special applications. This SR will serve as a reconnaissance robot for rescue services to survey the road tunnel during a traffic accident. More precisely, it is an SR with a direct-driving mechanism, which also involves a measuring unit that slides out of the robot shell. The sensor head, being mounted on the pull-out mechanism, is used to obtain data about the environment.

## 8. Conclusions

In most cases, mobile robots for special applications are either usually robust or small but without direct control. Our proposed mobile robot eliminates these disadvantages in that it combines these two categories of robots into one spherical robot.

Consequently, our proposed mobile robot will be used for reconnaissance in tunnels during an emergency. Secondly, writers achieved holonomy in terms of using the direct driving mechanism. Besides that, another advantage of using driving mechanism mentioned arises. To put it clearly, it is the direct connection of the internal control unit to the sensor unit.

Next, the Raspberry Pi 4 was designed as the main control unit. DC motors with 1150 RPM and 12 V voltage, as well as omnidirectional wheels with a diameter of 80, appeared appropriate for movement of the robot. Last but not least, motors are controlled by an MDD10A controller.

An overview of sensors suitable for our sensor unit has been created. The solutions we propose are as follows. A preference for a CO${}_{2}$ sensor was given to the MQ-135 sensor. The MQ-7 sensor will execute measurement of CO. Furthermore, K-type thermocouples will be used for temperature sensing. The selection of OV7670 and MLX90640 type cameras was made from the available papers. Finally, LiDAR RPLIDAR-A1 can be used as a scanning device for our robot.

As the robot is currently in the construction and design stage, it is not yet possible to publish the results of the corner measurements. The results will be then summed up in a following article.

## Figures and Tables

**Table 1:** An overview of applied SR mechanisms in existing papers.

Mechanism	Driving	Holonomy	Use
Single wheel	D	-	References [29,30,31]
Hamster ball	D	v*	References [32,33,34,35,36]
Mass movement	G	v	References [38,39,40]
Pendulum	G	v*	References [3,17,41,42,43,44,45,46,47]
Gimbal mechanism	G	v*	References [58,59,60,61,62,63]
Orthogonal flywheels	M	v*	References [67,69,70]
Flywheel on pendulum	M	-	References [71,72,73]
Deformation	O	v	References [74,75]
Hybrid	O	v*	References [76,77,78,79,80]

**Table 2:** Comparison of functional features of SRs driving mechanism.

Mechanism	CDoF	Mobility	Maneuve- Rability	Stability	Dead Reckoning	Design
Single wheel	2	Good	Weak	Weak	Weak	Simple
Hamster ball	2	Good	Weak	Great	Weak	Simple
Mass movement	3	Great	Good	Great	Good	Difficult
Pendulum	2*	Good	Great	Great	Great	Average
Gimbal mechanism	2*	Good	Good	Good	Good	Difficult
Orthogonal flywheels	2	Great	Good	Good	Good	Difficult
Flywheel on pendulum	3	Great	Great	Good	Good	Average
Deformation	3	Good	Good	Great	Weak	Difficult
Hybrid	2*	Good	Good	Great	Good	Average

**Table 3:** Review of cameras with main properties.

Spectrum	Label	Resolution [MP]	Pixels (W × H)	Maximum Image Rate [fps]	Power Consumption [mW]	Use
Visible	OV9655	1.3	1280 × 1024	15–30	90	References [103,104,119]
	OV7670	0.3	640 × 480	30	60	References [105,106,107,108,120]
	OV2640	2	1632 × 1232	15–60	125–140	References [109,110,121]
	MT9M001	1.3	1280 × 1024	30	325	References [111,112,122,123]
IR	AMG8833	64 × 10${}^{-6}$	8 × 8	1–40	15	References [113,114,115,124]
	MLX90640	764 × 10${}^{-6}$	32 × 32	64	83	References [116,117,118,125]

**Table 4:** Overview of the concentration and its consequences on the human body.

CO${}_{2}$		CO	
**Concentration [ppm]**	**Effect**	**Concentration [ppm]**	**Effect**
≤1000	None (standard air value)	100	Mild headache
1000–2000	Drowsiness, slight difficulties	200–300	Headache
2000–5000	Headache, loss of concentration	400–600	Severe headache, nausea
5000	Severe headache unconsciousness	1100–1500	Increased pulse, fainting, unconsciousness
≥40,000	Suffocation	5000–10,000	Weak pulse, respiratory arrest

**Table 5:** Review of gas sensors with main properties.

Gas	Label	Detection Range [ppm]	Power Consumption [mW]	Use
CO${}_{2}$	MQ-135	10–1000	≤950	References [127,128,130,131,134,135]
	MG811	350–10,000	850 ± 120	References [132,133,139,140,141]
	CCS811	400–32,768	60	References [136,137,138]
CO	MQ-7	20–2000	350	References [129,130,143,145,147,152]
	MQ-9	10–1000	≤350	References [142,144,153]

**Table 6:** Review of thermocouples with main properties.

Thermocouple	Temperature Range [°C]		Error [%]	Use
	Short Measurement	Long Measurement		
Type E	40–900	0–800	±0.50	Stronger signal in low-temperature range, more stable
Type J	−180–800	0–750	±0.75	Short lifespan in high-temperature range
Type K	−180–1300	0–1100	±0.75	Cheap, precise, reliable, wide temperature range
Type N	−270–1300	0–1100	±0.75	Expensive, more accurate in low-temperature range
Type R	−50–1700	0–1600	±0.25	Expensive, high precision and stability
Type S	−50–1750	0–1600	±0.25	Expensive, high precision and stability
Type T	−250–400	−185–300	±0.75	Extremely reliable in low-temperature range
Type B	0–1820	200–1700	±0.50	Stable and precise in extremely high-temperature range

## Data Availability

Not applicable.

## Abbreviations

The following abbreviations are used in this manuscript:

SR	Spherical robot
IDU	Internal Drive Unit
CoM	Center of Mass
CMG	Control Moment Gyroscopes
DoF	Degree of Freedom
CDoF	Control Degree of Freedom
ppm	parts per million
IoT	Internet of Things
AQI	Air Quality Index
ICU	Internal Control Unit
SU	Sensors Unit
